# Insect Herbivore Populations and Plant Damage Increase at Higher Elevations

**DOI:** 10.3390/insects12121129

**Published:** 2021-12-17

**Authors:** Sulav Paudel, Pragya Kandel, Dependra Bhatta, Vinod Pandit, Gary W. Felton, Edwin G. Rajotte

**Affiliations:** 1 Department of Entomology, The Pennsylvania State University, State College, PA 16802, USA; gwf10@psu.edu (G.W.F.); uvu@psu.edu (E.G.R.); 2 Microbial Solutions Team, AgResearch Ltd., Lincoln 7674, Christchurch 8140, New Zealand; 3 Bredesen Center for Interdisciplinary Research and Graduate Education, University of Tennessee, Knoxville, TN 37996, USA; Pkandel@vols.utk.edu; 4 Louisiana Department of Health, Northeast Delta Human Services Authority, Monroe, LA 71201, USA; Bhatta.dependra@gmail.com; 5 Plantwise, Center for Agriculture and Bioscience International (CABI), South Asia Office, New Delhi 110012, India; v.pandit@cabi.org

**Keywords:** climate change, elevational gradient, insect pests, Nepal, herbivory

## Abstract

**Simple Summary:**

It is vitally important to understand the effects of climate change on insect pest populations and crop losses. Using elevation as a proxy for climate change, a field study was conducted in farmer’s fields in Nepal at various elevations in the Himalayan Mountains. At higher elevations, natural herbivore populations and plant damage from herbivory were significantly higher compared to their low-elevation counterparts. Temperature varied with elevation in the field and significantly affected both insect populations and plant damage. A geographical shift of insect pests towards higher elevations is predicated, so it is important to better understand how biotic and abiotic ecological factors and evolutionary processes will act together to affect ecosystem dynamics to reliably predict future pest problems.

**Abstract:**

Elevation gradients are used as a proxy to simulate climate change effects. A field study was conducted along an elevational gradient in Nepal to understand the effects of abiotic conditions on agriculturally important insect herbivore populations (tobacco caterpillar: *Spodoptera litura*, tomato fruit worm: *Helicoverpa armigera*, and South American leaf miner, *Tuta absoluta*) and herbivory damage on tomatoes. Elevation ranged from 100 m to 1400 m above sea level, representing different climatic zones where tomatoes are grown. Contrary to our hypothesis, natural herbivore populations and herbivory damage significantly increased at higher elevations. Individual insect species responses were variable. Populations of *S. litura* and *T. absoluta* increased at higher elevations, whereas the *H. armigera* population was highest at the mid-elevational range. Temperature variations with elevation also affected insect catch numbers and the level of plant damage from herbivory. In the context of climate warming, our results demonstrate that the interactive effects of elevation and climatic factors (e.g., temperature) will play an important role in determining the changes in insect pest populations and the extent of crop losses.

## 1. Introduction

Elevational gradients are characterized by large variations in environmental conditions over relatively short distances [1]. Therefore, they are used in experiments to simulate changes expected under climate change scenarios [2]. Studies on how insect–plant interactions change with elevation are largely skewed towards woody and perennial herbaceous plants while impacts on agriculture crops are limited [3].

Crops are generally highly responsive to changes in both biotic and abiotic stressors [4,5]. Abiotic factors (e.g., temperature, CO2, UV radiation, air pressure) vary with elevation. In general, a low-level of herbivore abundance and damage is predicted at higher elevations due to harsher environmental conditions [6,7]. In addition, the level of plant secondary compounds (e.g., phenolics, glucosinolates, volatile organic compounds) and leaf morphological characteristics (e.g., leaf toughness and trichome density) can also vary with changing abiotic conditions affecting plant resistance to herbivores along elevation gradients [3,8,9]. A combination of herbivore pressure and plant defense responses can presage the level of plant damage and crop losses in the context of a warming climate [10]. Ambient temperature, which declines at higher elevations, is considered one of the most important factors affecting herbivore and plant traits [11,12].

There is limited knowledge of variation in insect populations and plant damage along elevational gradients in vegetable systems in Nepal. The present study evaluates the changes in natural herbivore populations and herbivory damage in tomatoes (*Solanum lycopersicum*) along an elevation gradient in Western Nepal. We tested the hypothesis that the extent of insect herbivory will decrease along an elevational gradient. Insect populations were monitored over time using pheromone traps, whereas data on plant damage were measured as a percentage of total leaves damaged from herbivory. Nepal is an ideal country for the research because it is highly diverse in terms of climate, ecosystems, and floral and faunal species; within a north–south trajectory of only 80 km, climatic regimes from the Florida Keys to the Arctic are found [13].

## 2. Materials and Methods

**Figure 1:**
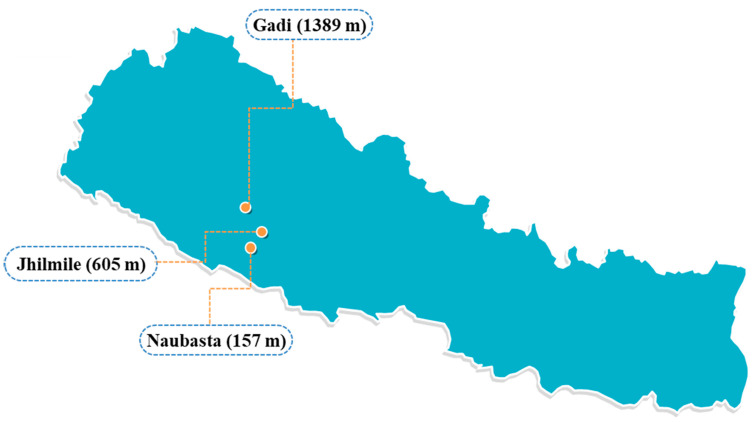
Research sites in Nepal—Naubasta, Banke (157 m; 28.2505° N, 81.6729° E), Jhilmile, Surkhet (605 m; 28.4398° N, 81.7363° E), and Gadi, Surkhet (1389 m; 28.6410° N, 81.6136° E).

The study was conducted in Western Nepal, on a south–north, low- to high-land transect that bridges the country’s physiographic regions, from Naubasta, Banke (157 m: 28.2505° N, 81.6729° E) to Gadi, Surkhet (1389 m: 28.6410° N, 81.6136° E), passing through Jhilmile, Surkhet (605 m: 28.4398° N, 81.7363° E) (Figure 1). The gradient spanned an elevation difference of 1232 m with vegetable farms throughout. At each elevation, three replicate tomato plots (50 m^2^ area) within a 500 m radius were established (Figure S1) on working smallholder farms. The field study was conducted from June to December 2018. Temperature was continuously monitored throughout the experiment using a temperature logger (HOBO MX2300 Temp, Bourne, MA, USA) in each of the three elevations (one per elevational site). Temperature loggers were fitted onto a 5 m-tall stick at a height of 3 m and left in the open sun in an appropriate location at the center of one of the farms. Temperature was recorded automatically every 30 min and weekly mean was used for data analysis.

Tomato is one of the most important vegetables in Nepal, the consumption of which has tremendously increased in recent times [14,15]. Insect herbivore species are key pests of tomatoes in Nepal [16,17]. We assessed leaf herbivory damage as well as populations of tomato fruit worm (*Helicoverpa armigera*), tobacco cutworm (*Spodoptera litura*), and South American tomato leaf miner (*Tuta absoluta*), weekly by monitoring them along an elevational gradient using pheromone traps and crop damage estimates. *H. armigera* and *S. litura* are polyphagous pests native to Asia and surrounding regions, whereas *T. absoluta* is one of the recent invasive pests into the region.

Tomato var. Srijana (Nakkhu Seeds, Kathmandu, Nepal) was used for the experiment, which is a locally obtained popular tomato variety and is recommended for regions up to 2200–2400 m.a.s.l in Nepal. Seedlings (*n* = 36/site/elevation) were transplanted in black plastic bags (12″ × 20″) with a common potting soilless mixture (vermicompost and sand) to mitigate soil effects. Seedlings were planted during June–July (spacing- Rows × Plants: 75 × 60 cm). Urea, diammonium phosphate (DAP) and murate of potash (MOP) were used at the rate of @ 200:140:140 kg/ha; 50% of urea and 100% of DAP and MOP were mixed with the common potting mixture and two split doses of remaining urea after 15 and 30 days of transplanting. Organic manure prepared from local farm wastes and crop residues was used @ 30,000 kg/ha.

Populations of tomato fruit worm (*H. armigera*), tobacco caterpillar (*S. litura*) and South American tomato leaf miner (*T. absoluta*) were monitored weekly using pheromone traps targeted for these insects (Pest control India, Bangalore, India, https://www.pestcontrolindia.com/products/ (accessed on 21 March 2018)). Funnel traps were used for *H. armigera* and *S. litura* and water traps for *T. absoluta*. The farm settlements were isolated, and the villages where the experiments were conducted were within a 1–2 km area; therefore, one trap per insect species per elevation provided sufficient coverage. Traps were hung in the middle of the research plot at about 2 m above the ground level. There was at least 5 m between traps. Synthetic pheromone lures (Pest control India, Bangalore, India https://www.pestcontrolindia.com/products/ (accessed on 7 April 2018)) for each individual species were replaced every two months as recommended. Traps were checked weekly. Individual target species were identified, and the number trapped was expressed as the average number of insects captured/species/week/ elevation. Trap counts for the three insect species were analyzed separately as well as grouped together to calculate total insects trapped/week/elevation. There were 16 observation dates.

Leaves with characteristic damage by leaf-feeding herbivores and leaf miners (e.g., holes, mines, chewing damage) were considered damaged leaves. Two weeks after transplantation, 10 plants/site/elevation were randomly selected and checked weekly for leaf damage. Two different approaches were used. First, binary data on whether a plant had been damaged or not was recorded by checking individual leaves from a plant for any kind of herbivore damage. Secondly, the percentage (%) leaf damage from herbivory was calculated by counting the number of damaged leaves relative to the total number of leaves from a plant [18,19]. Using the percentage leaf damage approach helps address potential variation due to plant size across sites.

*Statistical analysis*: The total number of insects trapped (*n*) per week in different elevations for individual species and the sum of all three species was analyzed with ‘elevation’ as categorical predictors via generalized linear model (GLM) with Poisson distribution [20]. Pair-wise comparisons between elevational sites were carried out using Tukey mean comparisons.

The correlation between the temperature and insect count data was analyzed first by calculating the cross-correlation of the paired time series. The cross-correlation was calculated simultaneously by assuming a non-delayed insect number response to the temperature. This was followed by shifting the insect count series by one lag (one time interval) at a time behind the temperature series to see if there was any delayed response, as indicated by the cross-correlation value larger than the simultaneous cross-correlation value. Once the maximum cross-correlation was achieved, either with or without the time shifting of the insect count series, the identified maximum cross-correlation between each paired series was analyzed in time series regression. The insect count number was the dependent variable, and the temperature was the explanatory variable.

The binary data (plants damaged vs. undamaged) were compared among elevation sites using a GLM with binomial distributions through a logit link function with ‘elevation’ as a single factor. Percentage leaf damage from herbivore data was arcsine transformed to ensure that the distribution of analyzed data was approximately normal. The data from the three elevations were analyzed using analysis of variance (ANOVA) with ‘elevation’ as a single factor and pair-wise comparisons were made using the Fisher’s least significant difference method.

Later, the arcsine-transformed damage data were analyzed by linear regression: Arcsine (Damage) = Elevation + Temperature + Elevation × Temperature, where ‘Elevation’ was a categorical variable representing three elevations while ‘Temperature’ was a numerical variable, and Elevation × Temperature was the interaction effect of the two. Consequently, three different regression lines were fitted in a single analysis, with each line representing the correlation between temperature and plant damage at each elevation. These single regression analyses also allowed statistical comparison between the three regression lines; thus, we tested the elevation-and-temperature interaction effects by comparing the slopes between the lines. Analyses were carried out using Minitab software [21].

## 3. Results

### 3.1. Temperature Variation with Elevation

As expected, the temperature was reduced at higher elevations (Figure S1). The mean air temperature from June to December 2018 declined as the elevation increased (i.e., from 25.7 °C at 157 m to 20.2 °C at 1389 m). The lowest temperature recorded at Gadi (1389 m) was 12.9 °C while the highest was 26.0 °C with a median temperature of 21.6 °C. In Jhilmile (605 m), the lowest temperature recorded was 15.0 °C while the highest was 29.7 °C with a median temperature of 25.8 °C. Similarly, the lowest temperature recorded in Naubasta (157 m) was 17.2 °C while the highest was 32.0 °C with a median temperature of 24.8 °C. Despite their average temperature of between 26.7 °C and 32.2 °C, the maximum temperature in Jhilmile and Naubasta consistently exceeded 37.8 °C during daytime (Figure S2).

### 3.2. Insect Herbivore Populations

**Figure 2:**
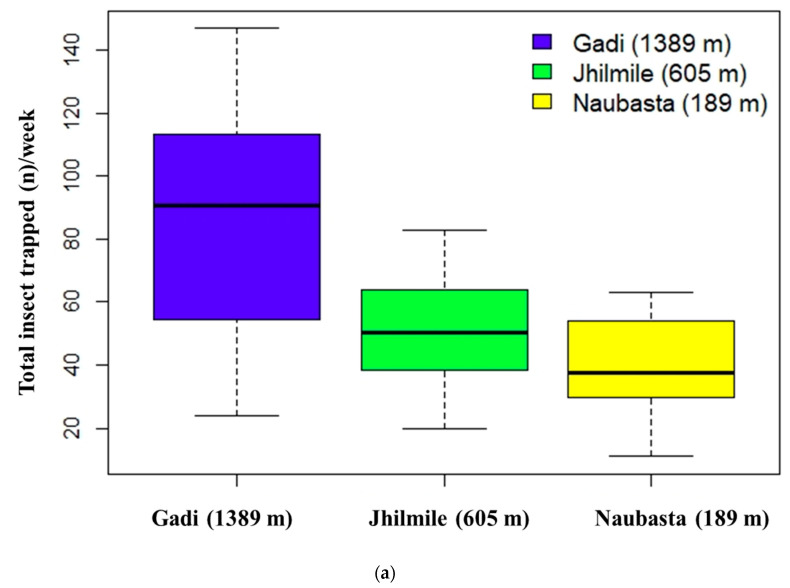

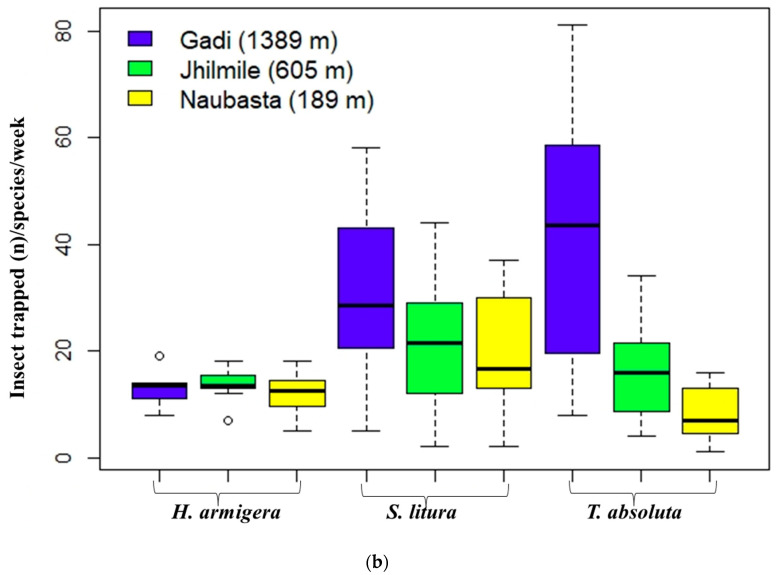
Insects trapped in pheromone traps at three different elevations: Naubasta (157 m), Jhilmile (605 m) and Gadi (1389 m); (**a**) box-plot diagram showing variation in average number of total insect trapped including *H. armigera*, *S. litura* and *T. absoluta* (total insect trapped (*n*)/week) and **(b**) box-plot diagram showing variation in the average number of insects trapped/week/elevation delineated by each individual herbivore species. The segment inside the rectangle represents the median insect trapped and “whiskers” above and below the box correspond to the minimum and maximum number of insects trapped. Circles (〇) indicate outliers.

The sum of three individual insect species (*H. armigera*, *S. litura*, and *T. absoluta*) trapped per week significantly varied with elevation (χ^2^ (2, 30) = 54.56, *p* < 0.01). The overall insect catch number in Gadi (1389 m) was significantly higher than the other two elevations (*p* < 0.000), but Jhilmile (605 m) and Naubasta (157 m) were not different (Figure 2a). The independent effect of elevation was also significant for *S. litura* (χ^2^ (2, 30) = 31.63, *p* < 0.01) and *T. absoluta* (χ^2^ (2, 30) = 17.53, *p* < 0.01) but not for *H. armigera* (χ^2^ (2, 30) = 3.00, *p* = 0.22). Average populations of *S. litura* and *T. absoluta* were highest at Gadi (1389 m) followed by Jhilmile (605 m) and Naubasta (157 m) (Figure 2b). The *H. armigera* population, however, was highest at Jhilmile (605 m) followed by Gadi (1389 m) and Naubasta.

The number of *S. litura* caught in pheromone traps and the sum of all three insect species were positively correlated with temperature in Gadi (1389 m) (Table 1). Other correlations were not significant.

### 3.3. Leaf Damage from Herbivory

**Figure 3:**
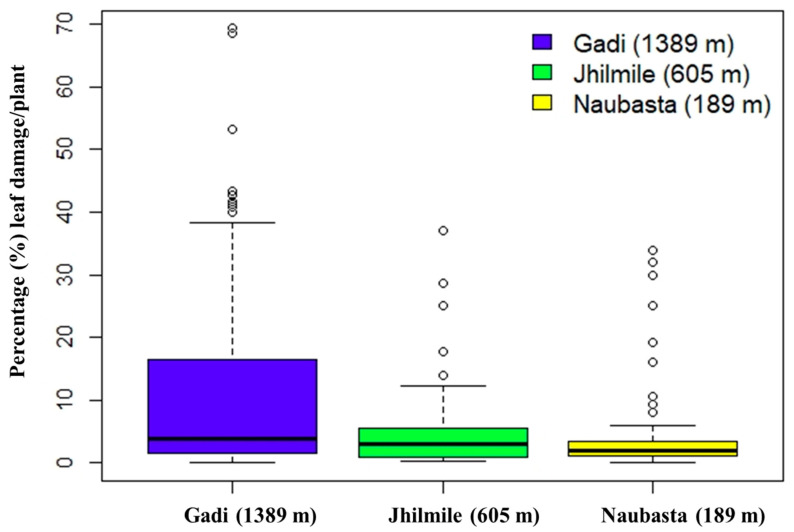
Box-plot diagram showing leaf damage by herbivores (%) in tomato plants from three different elevations: Naubasta (157 m), Jhilmile (605 m) and Gadi (1389 m). The segment inside the rectangle shows the median and “whiskers” above and below the box show the minimum and maximum damage (%), respectively. Circles (〇) symbolize outliers.

There were 915 observations (Gadi: 345, Jhilmile: 345, Naubasta: 225) over the entire study period, of which 299 plants had observable damage from herbivory. Approximately 6% of plants died during the experiment. There was a significant effect of elevation on the percentage of leaves damaged (F _(2,296)_ = 13.37, *p* < 0.001) but not for the number of plants damaged vs. undamaged. Based on pair-wise comparisons, plants at Gadi (1389 m) had a significantly higher proportion of leaf damage per plant than Naubasta (189 m) and Jhilmile (605 m) (*p* < 0.001) (Figure 3). Level of damage between Naubasta (189 m) and Jhilemile (605 m) was not significantly different.

**Figure 4:**
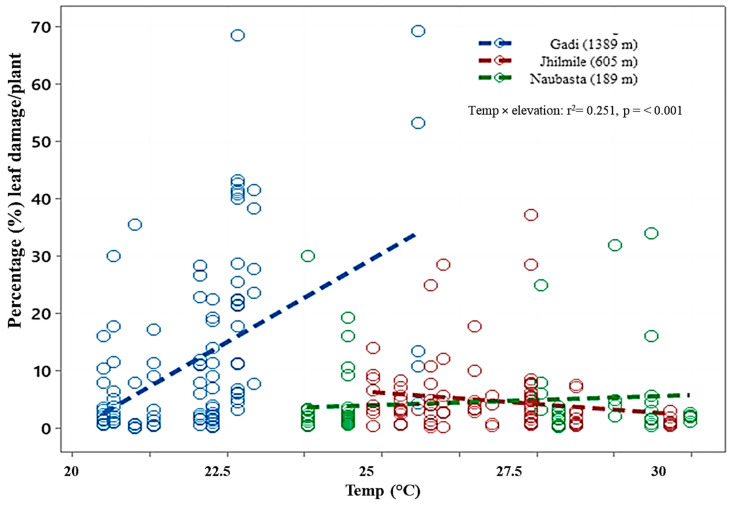
Scatterplot showing correlations between temperature and plant damage (leaf damage by herbivores/plant (%) in three different elevations: Gadi (1389 m), Jhilmile (605 m) and Naubasta (157 m). r^2^ coefficient and associated *p*-values are shown. Each circle within the plot represents % of leaves damaged by herbivores per plant per week. Different colors represent different elevation sites (blue: Gadi; red: Jhilmile, green: Naubasta).

There was a significantly positive relationship between temperature and level of plant damage in Gadi (1389m), but they were negatively correlated in Jhilmile (605 m) (Table 2). When compared among elevations, the temperature–plant-damage relationship in Gadi (1389 m) was significantly different (*p* < 0.001) from Jhilmile (605 m) and Naubasta (189 m). In addition, Jhilmile (605 m) was also statistically (*p* = 0.033) different from Naubasta (189 m). The elevation-and-temperature interaction effect was statistically significant (F _(2,293)_ = 27.65, *p* < 0.001) (Figure 4 and Table 2).

## 4. Discussion

Insect herbivore populations and level of plant damage from herbivory increased with elevation. Comparable results were reported for Antesia Bug, *Antestiopsis thunbergia*, in Arabica coffee in Tanzania, where the population density of the insects increased with elevation, (1000–1700 m a.s.l) [22]. Herbivore populations and plant damage in Ficus spp. (200 m to 2700 m a.s.l) were also significantly higher at a mid-elevation range (700 to 1200 m a.s.l), which is comparable to our experimental elevation range (605 to 1389 m a.s.l) [23]. These results, however, contrast with the general prediction of greater herbivore damage in plants growing closer to sea level because of a warmer climate [7,24]. However, the generality of this prediction should be interpreted with caution. For example, a correlation between elevational gradients and herbivory was not found in non-woody species in a global analysis of insect herbivory with 1027 plant species [11]. Similarly, Moriera et al. in their review also questioned the hypothesis, providing evidence of several interactions including positive, negative and non-linear associations with elevation [3]. These discrepancies in the findings may have resulted from a large variation in biotic and abiotic factors along an elevational gradient and their independent and combined influences on insect–plant interactions [3,11,25]. Paudel et al. termed this phenomenon an ‘asymmetric response to climate warming’ [26].

Temperature variations within the elevation gradient affected natural herbivore levels and plant damage. This result has important ecological consequences in the context of climate change. An increase in herbivore abundance, as well as a geographical shift of insect pests towards higher elevations, is expected with rising temperature [24,27]. Temperatures in tropical regions for most insects are already in an optimum range; therefore, any further increases may significantly reduce their abundance and diversity [28]. At the same time, increasing temperatures at higher latitudes or temperate regions might benefit from a northward range expansion resulting in higher insect abundance and crop losses [29,30]. Therefore, the potential of insect pests to better adapt will determine species distribution and community interactions in the face of climate change.

We also observed asymmetric elevation-specific variations in individual insect herbivore populations. This may be due to the patterns of temperature changes in these three elevational sites as well as the cropping system. The temperature in Naubasta (157 m) and Jhilmile (605 m) went consistently over 37.8 °C, which is considered a critical temperature threshold for several insect activities: 35.9 °C to 38.5 °C for *T. absoluta* [31], 38.0 °C for *S. litura* [32], >37.0 °C for *H. armigera* [33]. In contrast, the high-elevation site (Gadi, 1389 m) experienced comparatively moderate conditions. Furthermore, tomato production in Nepal is staggered in time, depending on elevation. In the hills (500 to 2000 m), tomatoes are successfully produced year round under plastic houses with open sides [34], which may provide food and shelter to insect herbivores specializing on tomatoes. At lower elevations (<500 m), the production window is limited (Aug–Feb) and is constrained by hot temperatures, low fruit set/flowering and diseases.

## 5. Conclusions

Overall, the present study suggests that natural herbivore populations and plant damage increase at higher elevations and are influenced by abiotic factors (e.g., temperature). While this study was focused on a single elevational gradient, the results are indicative of some of the changes in insect pest populations and plant damage with global warming. The asymmetric effect of site-specific temperature variations on individual insect pests is indicative of the complexity involved in predicting the climate-change-mediated effects on insect–plant interactions. Future studies including broader and multiple elevational ranges with a complete set of insect pests associated with a crop are necessary to bolster the results and conclusions drawn from this study.

## Figures and Tables

**Table 1:** Regression analysis of cross-correlation between temperature and insect catch number in different elevational sties.

Elevation Site	Insect Species	Time Series Regression Equation	Slope (Mean ± SE)	Intercept	r^2^ Value	F-Statistics	*p*-Value
Gadi (1389)	*H. armigera + S. litura + T. absoluta*	Trap catch number = −357.40 + 19.331 × Temperature	19.331 ± 7.274	−357.40 ± 167.10	0.414	F _(1,10)_ = 7.06	0.024
Jhilmile (605 m)	*H. armigera + S. litura + T. absoluta*	Trap catch number = 190.90 − 5.105 × Temperature	−5.105 ± 6.453	190.90 ± 176.00	0.073	F _(1,8)_ = 0.63	0.452
Naubasta (189 m)	*H. armigera + S. litura + T. absoluta*	Trap catch number = −63.88 + 3.621 × Temperature	3.621 ± 1.730	−63.88 ± 49.13	0.354	F _(1,8)_ = 4.38	0.070
Gadi (1389)	*H. armigera*	Trap catch number = −9.36 + 0.982 × Temperature	0.982 ± 0.818	−9.36 ± 18.80	0.126	F _(1,10)_ = 1.44	0.258
Jhilmile (605 m)	*H. armigera*	Trap catch number = 27.58 − 0.516 × Temperature	−0.516 ± 0.684	27.58 ± 18.48	0.054	F _(1,10)_ = 0.57	0.469
Naubasta (189 m)	*H. armigera*	Trap catch number = 30.83 − 0.668 × Temperature	−0.668 ± 0.432	30.83 ± 12.33	0.193	F _(1,10)_ = 2.39	0.153
Gadi (1389)	*S. litura*	Trap catch number = −167.86 + 8.681 × Temperature	8.681 ± 3.063	−167.86 ± 70.38	0.445	F _(1,10)_ = 8.03	0.0018
Jhilmile (605 m)	*S. litura*	Trap catch number = −31.45 + 1.987 × Temperature	1.987 ± 3.647	−31.45 ± 98.86	0.032	F _(1,10)_ = 0.30	0.599
Naubasta (189 m)	*S. litura*	Trap catch number = −45.45 + 2.262 × Temperature	2.262 ± 1.082	−45.45 ± 30.73	0.353	F _(1,8)_ = 4.37	0.070
Gadi (1389)	*T. absoluta*	Trap catch number = −180.20 + 9.669 × Temperature	9.669 ± 5.520	−180.20 ± 126.80	0.235	F _(1,9)_ = 3.07	0.110
Jhilmile (605 m)	*T. absoluta*	Trap catch number = 98.44 − 3.040 × Temperature	−3.040 ± 2.751	98.44 ± 75.01	0.132	F _(1,10)_ = 0.30	0.599
Naubasta (189 m)	*T. absoluta*	Trap catch number = −20.10 + 1.004 × Temperature	1.004 ± 0.533	−20.10 ± 15.12	0.308	F _(1,8)_ = 3.56	0.096

**Table 2:** Elevation-level-specific relationships between temperature (°C) and arcsine-transformed leaf damage percentages estimated from single regression analysis with r^2^ value.

Gadi (1389 m)	
Equation	Arcsine(Damage) = − 120.323 + 6.005 × Temperature
Slope	6.005 ± 0.769 (mean ± SE); *F* = 60.91, df = 1, 293, *p* < 0.001
Intercept	−120.323 ± 17.532 (mean ± SE)
**Jhilmile (605 m)**	
Equation	Arcsine(Damage) = 47.173 − 1.330 × Temperature
Slope	−1.330 ± 0.675 (mean ± SE); *F* = 3.88, df = 1, 293, *p* = 0.050
Intercept	47.173 ± 18.399 (mean ± SE)
**Naubasta (189 m)**	
Equation	Arcsine(Damage) = −1.956 + 0.457 × Temperature
Slope	0.457 ± 0.489 (mean ± SE); *F* = 0.87, df = 1, 293, *p* = 0.351
Intercept	−1.956 ± 13.148 (mean ± SE)
r^2^ = 0.251	

## Data Availability

Data is contained within the article and supplementary material.

## Supplementary Materials

The following are available online at https://www.mdpi.com/article/10.3390/insects12121129/s1, Figure S1: Average air temperatures from June to December 2018 in three different elevations, Naubasta (157 m), Jhilmile (605 m) and Gadi (1389 m); Figure S2: Variation in air temperatures from May to Nov 2018 in three different elevations, Gadi (1389 m), Jhilmile (605 m) and Naubasta (157 m) as recorded by the temperature logger.
