# Supplementary material for: Insect Herbivore Populations and Plant Damage Increase at Higher Elevations
Source: Insects. 2021 Dec 17;12(12):1129. doi: 10.3390/insects12121129 (PMC8708097; doi:10.3390/insects12121129)
Supplement: Supplementary file 1 [file insects-12-01129-s001.zip › insects-1438200-supplementary.pdf]

insects MDPI

Supplementary Materials

Insect Herbivore Populations and Plant Damage Increase at Higher Elevations

Sulav Paudel, Pragya Kandel, Dependra Bhatta, Vinod Pandit, Gary W. Felton and Edwin G. Rajotte

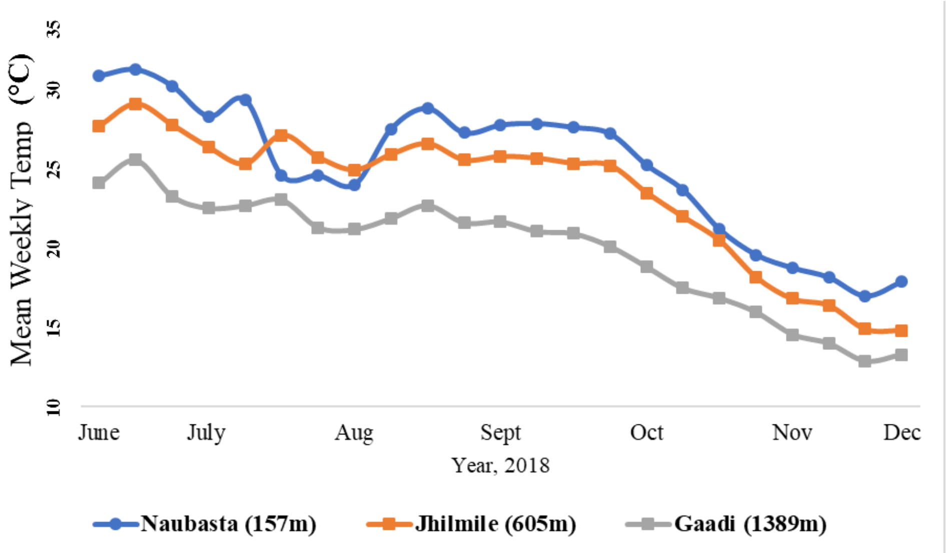

Figure S1. Average air temperatures from June to Dec 2018 in three different elevations, Naubasta (157 m), Jhilmile (605 m) and Gadi (1389 m).

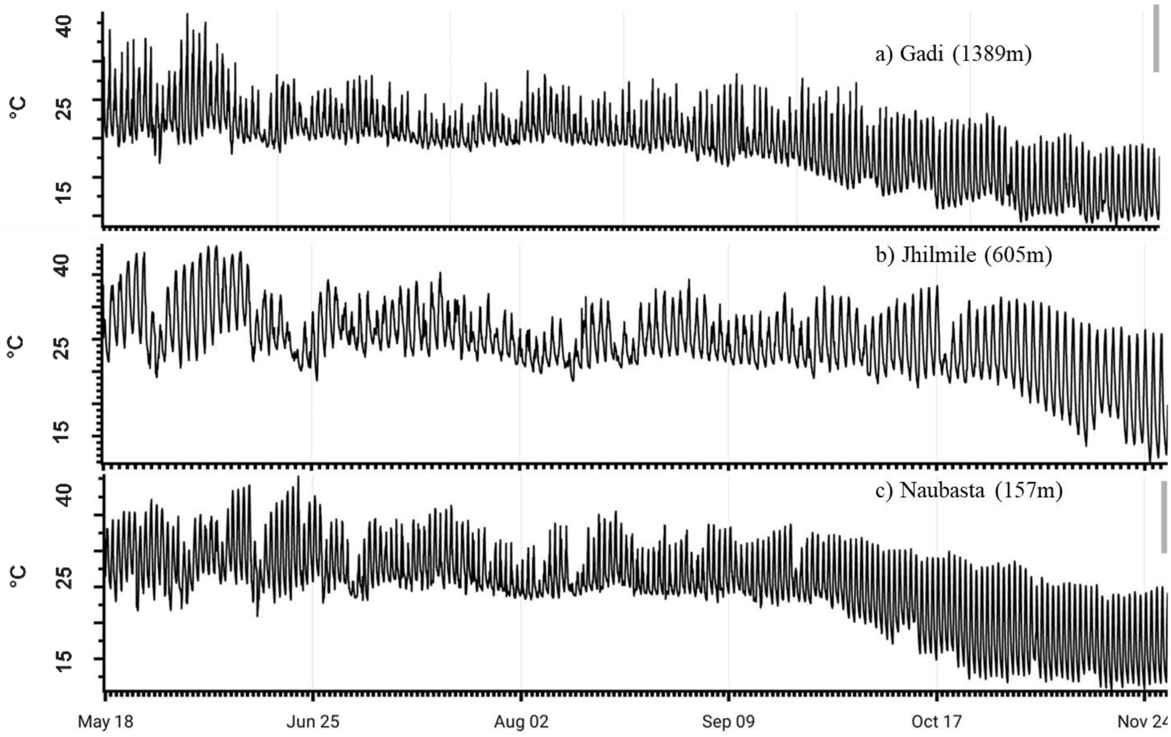

Figure S2. Variation in air temperatures from May to Nov 2018 in three different elevations, Gadi (1389 m), Jhilmile (605 m) and Naubasta (157 m) as recorded by the temperature logger.
